# Predictors of initiating high-efficacy vs. platform therapies as first-line disease-modifying treatment in multiple sclerosis

**DOI:** 10.3389/fneur.2026.1738815

**Published:** 2026-01-30

**Authors:** Khalid Bin Aziz, Thamer S. Alhowaish, Feras Alanazi, Mezyed Alghanim, Saleh Alkhreigi, Fahad Almubarak, Faisal Aleisa, Nouf AlTuwaijri, Raghad Alanazi, Yaser Al Malik

**Affiliations:** 1 College of Medicine, King Saud bin Abdulaziz University for Health Sciences, Riyadh, Saudi Arabia; 2 King Abdullah International Medical Research Center, Riyadh, Saudi Arabia; 3 Department of Neurology, King Abdulaziz Medical City, Ministry of National Guard Health Affairs, Riyadh, Saudi Arabia; 4 College of Medicine, Princess Norah University, Riyadh, Saudi Arabia; 5 College of Medicine, Northern Border University, Arar, Saudi Arabia

**Keywords:** multiple sclerosis, disease-modifying therapy, high-efficacy disease-modifying therapy, first-line treatment, treatment persistence, Expanded Disability Status Scale (EDSS), predictors, Saudi Arabia

## Abstract

**Background:**

The therapeutic landscape of multiple sclerosis (MS) is rapidly evolving, with increasing emphasis on early initiation of high-efficacy disease-modifying therapies (heDMTs). However, real-world data on predictors of first-line heDMT use and its outcomes remain scarce in the Middle East.

**Objectives:**

To identify demographic and clinical factors influencing the choice of first-line heDMT vs. platform therapy in relapsing-remitting MS (RRMS) and to evaluate whether initial treatment class impacts disability outcomes and treatment persistence.

**Methods:**

We conducted a retrospective cohort study of 826 RRMS patients treated at a tertiary center in Saudi Arabia between 2016 and 2024. Predictors of initiating heDMT were identified using multivariable logistic regression. Disability status (EDSS category) was analyzed using ordinal logistic regression. Treatment persistence and causes of discontinuation were examined using Kaplan–Meier survival analysis, Cox proportional hazards modeling, and Fine–Gray competing-risks regression.

**Results:**

Of 826 patients, 330 (40%) received heDMTs as first-line therapy. Initiation in the later treatment era (2019–2024) strongly predicted heDMT use compared with the early treatment era (2008–2013; OR = 48.8; *p* < 0.001), as did cerebellar symptom onset (OR = 2.51; *p* = 0.025). Age, sex, and comorbidities were not significant predictors. Starting on a heDMT did not translate into higher or lower disability levels compared with platform therapies (OR = 1.08; *p* = 0.767). However, patients initiating heDMTs demonstrated superior treatment persistence (12-month persistence: 91.7 vs. 83.4%; *p* < 0.0001) and a markedly lower risk of discontinuation (HR = 0.41; *p* < 0.001). Competing-risks analysis showed that heDMT users were significantly less likely to discontinue due to inefficacy or adverse events, but more likely to stop for other reasons, such as pregnancy, preference, or supply issues.

**Conclusions:**

First-line use of heDMTs in Saudi Arabia has increased substantially over recent years, particularly among patients with cerebellar onset. While disability outcomes were similar between treatment groups, initiating heDMTs conferred clear advantages in persistence and tolerability. These findings reinforce the paradigm shift toward early intensive MS management and highlight the need for broader access and guideline-driven implementation of heDMTs in the region.

## Introduction

Multiple sclerosis (MS) is a chronic immune-mediated demyelinating disease of the central nervous system and the most common cause of neurological disability in young adults worldwide (1). Its burden is rising in the Middle East; in Saudi Arabia, the prevalence of MS has increased to an estimated ~62 per 100,000 population (2). The introduction of disease-modifying therapies (DMTs) over the past three decades has revolutionized MS management by reducing relapse rates and slowing the progression of disability in relapsing forms of the disease (1).

Despite these therapeutic advances, the optimal strategy for initial DMT selection remains a subject of debate (3). Traditionally, MS has been managed with a stepwise “escalation” approach in which patients are started on platform therapies and switched to a more potent agent only if disease activity persists. In recent years, however, accumulating evidence has prompted a paradigm shift toward early use of high-efficacy disease-modifying therapies (heDMTs) as first-line treatment in appropriate patients, consistent with the “hit early, hit hard” strategy (4). Several highly effective monoclonal antibody and oral agents (e.g., natalizumab, ocrelizumab, alemtuzumab, cladribine, and sphingosine-1-phosphate receptor modulators) have demonstrated superior suppression of relapses and new lesions compared to platform therapies, albeit with increased risks and monitoring requirements (4). The rationale for an “induction” or early intensive approach is supported by emerging data showing that initiating heDMTs at disease onset can improve long-term outcomes. For example, a recent meta-analysis of seven studies found that over a 5-year horizon, patients started on early intensive therapy had ~30% lower risk of disability worsening (measured by confirmed Expanded Disability Status Scale (EDSS) progression) than those managed with an escalation strategy (5). Likewise, an observational cohort reported that initial treatment with heDMTs (such as fingolimod, natalizumab, or alemtuzumab) was associated with a significantly reduced risk of conversion to secondary progressive MS compared to starting on interferon or glatiramer acetate (6).

Nevertheless, real-world practice shows considerable variation in the uptake of heDMTs as first-line therapy. Clinicians must balance the greater efficacy of heDMTs against their potential adverse effects, costs, and patient-specific factors. Recent evidence indicates that the decision to start a patient on heDMT is influenced by both clinical and demographic factors. For example, one large retrospective study found that only about one in four newly treated MS patients in the United States initially received a heDMT, and those who did were more likely to be male and to have early signs of aggressive disease (such as impaired ambulation or other disability requiring symptomatic treatments) (7). In contrast, patients presenting with primarily sensory or visual symptoms were significantly less likely to be started on a heDMT (7). These patterns suggest that physicians often reserve first-line heDMT for cases perceived as high-risk, while adopting a more cautious approach in patients with ostensibly milder disease.

In Saudi Arabia, data on MS treatment patterns and the determinants of initial therapy choice remain limited (1). There is some concern that the early use of heDMTs may be underutilized in our setting (8), possibly reflecting differences in practice preferences, resource availability, or risk aversion. Understanding how and why clinicians decide to employ heDMTs as first-line treatment in the local context is important, especially since early intensive treatment has been linked to improved patient outcomes (8). Another practical consideration is treatment persistence: heDMTs can offer durable disease control but may be discontinued due to safety/tolerability issues, whereas platform therapies might be switched early because of inadequate efficacy. Real-world studies have shown that roughly one-third of DMT discontinuations are due to adverse events and another third is due to lack of efficacy, and that after 10 years, over 80% of patients started on interferon or glatiramer acetate have switched therapies compared to only ~20% of those treated with alemtuzumab (9). These findings highlight the need to evaluate how initial DMT choice impacts long-term adherence and disease stabilization.

In this retrospective cohort study, we aimed to identify the determinants of first-line use of heDMTs in MS patients in Saudi Arabia, and to examine the clinical outcomes associated with initial therapy selection. Specifically, our primary objectives were to (1) determine which demographic and disease-related factors are associated with the decision to start a heDMT vs. a platform therapy as the first DMT, and (2) assess whether the category of first-line DMT is linked to patients' current disability status (Expanded Disability Status Scale score). In addition, we evaluated secondary outcomes of treatment persistence on the initial DMT, including the time to discontinuation of the first therapy and the reason for discontinuation (inefficacy, adverse events, or other factors). By elucidating these patterns, our study provides real-world evidence to inform early treatment strategies and optimize MS management in Saudi Arabia.

## Methods

### Study design and participants

This study was a retrospective cohort analysis of patients with MS treated at a tertiary care center in Saudi Arabia. We included all adult patients (aged ≥18) who had a confirmed relapsing-remitting MS (RRMS) diagnosis who were seen during the study period (March 2016 to August 2024). This included both patients who initiated their first DMT during this period and patients who had started a DMT prior to 2016 but continued follow-up and treatment during the study period. Patients with a diagnosis of secondary progressive or primary progressive MS and who had never started a DMT were excluded from the analysis. A total of 826 MS patients met the inclusion criteria. The cohort was followed from the start of the first DMT until treatment discontinuation or the end of the study period. The study was approved by the relevant institutional ethics committee, with a waiver of informed consent due to the retrospective use of de-identified patient data.

### Data collection and definitions

Demographic and clinical baseline data were collected from medical records, including age, sex, BMI, comorbidities (e.g. diabetes, hypertension, thyroid disease, and depression), diagnosis date, family history, initial clinical presentation, treatment information (e.g. first DMT type, start and discontinuation dates, and reason for discontinuation). Neurologic disability was assessed using the EDSS. For analysis, EDSS scores at the last follow-up (i.e., at data cutoff) were categorized into three disability levels: mild (EDSS 0–3.5), moderate (EDSS 4.0–5.5), and severe (EDSS ≥6.0). These categories were selected to reflect clinically meaningful disability thresholds commonly used in MS research, distinguishing patients with mild disability or no impairment (scores 0–3.5), moderate disability (ambulatory without continuous assistance, EDSS in the mid-range), and severe disability (significant impairment requiring assistance or wheelchair, EDSS 6 and above) (10).

All recorded DMTs were tabulated for each patient, including the start date of the first DMT, discontinuation (stop) date, and the documented reason for discontinuation (if applicable). For the purpose of analysis, we classified all DMTs into two therapeutic categories: heDMTs vs. platform therapies. In line with prior literature, the platform therapies were defined to include the traditional first-line injectable DMTs (interferon-β1a/1b and glatiramer acetate) as well as oral agents of moderate efficacy such as dimethyl fumarate and teriflunomide. The interferon-β1a formulations used in our cohort included Avonex, Rebif, and Plegridy, while interferon-β1b was represented by Betaseron; in cases where the specific interferon type or regimen could not be determined from the medical records, patients were categorized under “interferon beta (non-specific).” heDMTs were defined to include the more potent DMTs, principally the monoclonal antibody treatments (natalizumab, alemtuzumab, ocrelizumab, ofatumumab, and the off-label anti-CD20 antibody rituximab) and other highly active agents like cladribine and fingolimod (10). For patients with multiple sequential DMTs, the first DMT initiated was identified and categorized as heDMT or platform according to the above definitions. Additionally, the calendar year of first DMT initiation was stratified into discrete time periods (“eras”) to reflect changes in therapy availability over time. Specifically, first DMT start dates were grouped into three eras (2008–2013, 2014–2018, and 2019–2024) for use in subsequent analyses, to account for the evolving treatment landscape and shifting prescribing practices across these periods.

Treatment persistence was defined as the time from initiation of the first DMT until its discontinuation for any reason. Patients were monitored for discontinuation of the first DMT through regular clinic visits and pharmacy records. If a patient's first DMT was stopped, the discontinuation date and the reason for discontinuation (as documented by the treating neurologist) were recorded. If a patient had no recorded discontinuation date for the first DMT, the patient was assumed to be still ongoing with that therapy at the time of data cut-off; such patients were considered still on their initial DMT as of the data extraction date and were censored at that date in the persistence analysis. In this study, August 2024 served as the data cut-off for censoring ongoing first DMT treatments.

As part of data cleaning, the reasons for first DMT discontinuation were standardized into broader categories. Free-text reasons in the medical records were mapped to a set of predefined groups agreed upon by the investigators. Specifically, any reason indicating lack of efficacy or disease breakthrough (e.g. “failure/non-response” or “clinical/radiological progression”) was classified under inefficacy. Reasons related to medication intolerance or adverse events (such as “side effects” or “poor tolerance”) were grouped as adverse effects. Patient-driven or elective reasons were also consolidated: instances of pregnancy or family planning leading to therapy interruption were noted as a distinct category given their clinical importance, while other patient choice reasons (e.g. “patient preference to stop or switch”) and external factors (including drug unavailability) were categorized under patient preference/other. If no specific reason was documented or the reason was unknown, it was classified as unknown. This recategorization ensured consistency in analysis of discontinuation causes.

All study data were extracted and verified by the research team, and quality checks were performed to resolve any inconsistencies (for example, clarifying ambiguous drug names or partial dates by reviewing source records). All variables were then coded according to the definitions above for use in subsequent analyses.

### Statistical analysis

Descriptive statistics were used to summarize the baseline characteristics of the cohort. Categorical variables were presented as frequencies and percentages, while continuous variables were reported as medians with interquartile ranges (IQR). Differences between patients who initiated platform therapies vs. heDMTs were assessed using Pearson's Chi-squared test for categorical variables and the Wilcoxon rank sum test for continuous variables. Multivariable logistic regression analysis was performed to identify factors independently associated with the use of heDMT as the first-line treatment. Ordinal logistic regression was used to examine the association between baseline factors, including treatment category, and current disability status measured by the EDSS. Treatment persistence was evaluated using Kaplan-Meier survival analysis with group comparisons conducted via the log-rank test. A Cox proportional hazards model was used to estimate the hazard of treatment discontinuation, while competing-risks regression using the Fine–Gray method was applied to assess the subdistribution hazard ratios (sHR) for specific causes of discontinuation, including inefficacy, adverse events, and other reasons. Time from MS diagnosis to initiation of the first disease-modifying therapy was included as a continuous covariate in all multivariable models to account for potential confounding related to treatment timing. All analyses were conducted using RStudio (version 2024.9.1.394, Boston, MA, USA) with R version 4.4.2. A two-sided *p*-value of < 0.05 was considered statistically significant.

## Results

### Baseline characteristics

Among the 826 patients included in the study, the majority were female (67.4%), with a median age of 35.0 years (IQR = 30.0–42.0). Most patients had mild disability at the time of last follow-up, as measured by EDSS 0–3.5, accounting for 81.1% of the cohort. The distribution of BMI categories was relatively balanced, with 35.0% of patients classified as normal weight, 32.3% overweight, and 28.1% obese. Regarding comorbidities, 5.0% had diabetes mellitus and 4.7% had hypertension. A family history of MS was reported in 14.1% of cases, while 29.4% had unknown family history status. The most common initial clinical presentation was sensory symptoms (33.5%), followed by visual symptoms (23.8%). The median time from MS diagnosis to DMT initiation was 1.1 months (IQR = 0.0–12.2). Most patients initiated their first DMT in the 2019–2024 era (47.2%), followed by 2014–2018 (31.0%) and 2008–2013 (21.8%, Table 1).

**Table 1:** Baseline characteristics by first-line therapy.

**Characteristic**	**Missing**	**Overall *N* = 826**	**Platform *N* = 496**	**heDMT *N* = 330**	***p*-Value**
**Gender**					
Male	1 (0.1%)	269 (32.6%)	152 (30.7%)	117 (35.5%)	0.154
Female		556 (67.4%)	343 (69.3%)	213 (64.5%)	
Age (years)	0 (0%)	35.0 (30.0–42.0)	38.0 (32.0–44.0)	31.5 (27.0–37.0)	< 0.001
**BMI**					
Underweight	17 (2.1%)	38 (4.7%)	23 (4.7%)	15 (4.6%)	0.222
Normal		283 (35.0%)	169 (34.8%)	114 (35.2%)	
Overweight		261 (32.3%)	168 (34.6%)	93 (28.7%)	
Obese		227 (28.1%)	125 (25.8%)	102 (31.5%)	
Diabetes mellitus	4 (0.5%)	41 (5.0%)	32 (6.5%)	9 (2.7%)	0.015
Hypertension	2 (0.2%)	39 (4.7%)	29 (5.9%)	10 (3.0%)	0.060
Depression	3 (0.4%)	119 (14.5%)	84 (17.0%)	35 (10.6%)	0.011
Thyroid disease	2 (0.2%)	58 (7.0%)	40 (8.1%)	18 (5.5%)	0.146
**Family history of MS**					
No	2 (0.2%)	466 (56.6%)	272 (55.1%)	194 (58.8%)	0.555
Yes		116 (14.1%)	73 (14.8%)	43 (13.0%)	
Unknown		242 (29.4%)	149 (30.2%)	93 (28.2%)	
**Initial clinical presentation**					
Sensory	112 (14%)	239 (33.5%)	128 (31.0%)	111 (36.9%)	0.138
Motor		151 (21.1%)	100 (24.2%)	51 (16.9%)	
Cerebellar		70 (9.8%)	42 (10.2%)	28 (9.3%)	
Brainstem		71 (9.9%)	40 (9.7%)	31 (10.3%)	
Visual		170 (23.8%)	98 (23.7%)	72 (23.9%)	
Others		13 (1.8%)	5 (1.2%)	8 (2.7%)	
Time between MS diagnosis to initiation of therapy (months)	163 (20%)	1.1 (0.0–12.2)	0.0 (0.0–11.3)	3.0 (0.3–14.2)	< 0.001
**Era of DMT initiation**					
2008–2013	146 (18%)	148 (21.8%)	138 (37.0%)	10 (3.3%)	< 0.001
2014–2018		211 (31.0%)	164 (44.0%)	47 (15.3%)	
2019–2024		321 (47.2%)	71 (19.0%)	250 (81.4%)	
**EDSS**					
Mild	0 (0%)	670 (81.1%)	385 (77.6%)	285 (86.4%)	0.006
Moderate		70 (8.5%)	48 (9.7%)	22 (6.7%)	
Severe		86 (10.4%)	63 (12.7%)	23 (7.0%)	

n (%); median (Q1–Q3).

Pearson's Chi-squared test; Wilcoxon rank sum test.

### Characteristics of baseline therapies

**Figure 1:**
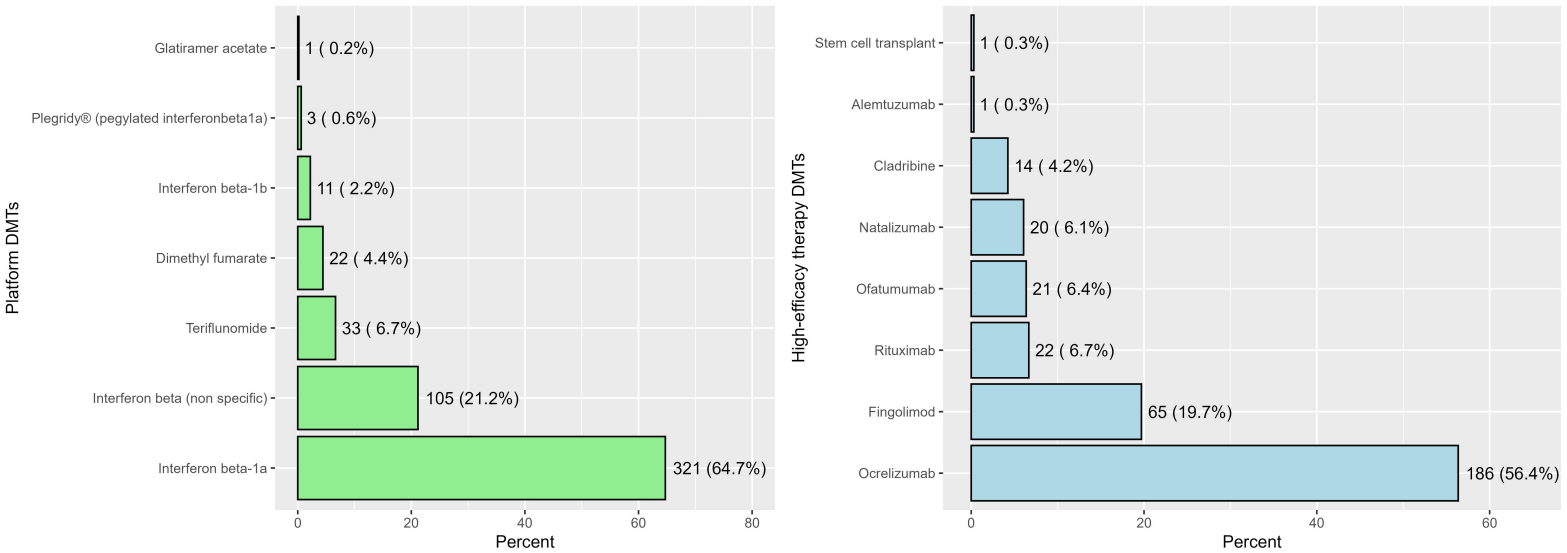
The frequencies and proportions of interventions across platform DMTs (*n* = 496) and heDMTs (*n* = 330).

Among patients under study, platform therapies were initiated in 496 patients, representing 60.0% of the total cohort, whereas heDMTs were initiated in 330 patients, accounting for 40.0% of the cohort. Among patients who received platform therapies (*n* = 496), the most frequently prescribed agent was interferon beta-1a, used in 321 patients (64.7%), followed by non-specific interferon beta formulations in 105 patients (21.2%). In the heDMT group (*n* = 330), ocrelizumab was the most used first-line agent, accounting for 186 patients (56.4%), followed by fingolimod in 65 (19.7%), and rituximab in 22 (6.7%, Figure 1).

### Statistical differences between platform and heDMT groups in terms of patients' characteristics

Significant differences were observed between the platform and heDMT groups across several baseline characteristics. Patients receiving heDMTs were younger (median age 31.5 years, IQR 27.0–37.0) compared with those receiving platform therapies (median 38.0 years, IQR 32.0–44.0; *p* < 0.001). The prevalence of diabetes mellitus (2.7 vs. 6.5%; *p* = 0.015) and depression (10.6 vs. 17.0%; *p* = 0.011) was lower among patients on heDMTs. The time from diagnosis to therapy initiation was longer in the heDMT group (median 3.0 months, IQR 0.3–14.2) than in the platform group (median 0.0 months, IQR 0.0–11.3; *p* < 0.001). Differences were also noted in disability status (EDSS category; *p* = 0.006), with a higher proportion of patients with mild disability initiating heDMT (86.4 vs. 77.6%).

A significant shift in prescribing patterns was observed over time (*p* < 0.001). Most patients who received platform therapies initiated treatment in earlier periods (37.0% between 2008–2013 and 44.0% between 2014–2018), whereas only 19.0% began in the most recent era (2019–2024). In contrast, the majority of patients initiating heDMTs (81.4%) did so during 2019–2024, reflecting a clear temporal trend toward earlier adoption of heDMTs (Table 1).

### Determinants of initiating heDMTs

In the multivariable logistic regression model, treatment initiation in more recent eras was independently associated with a higher likelihood of receiving heDMT as first-line treatment. Compared with patients who began therapy between 2008 and 2013, those initiating treatment from 2014 to 2018 had significantly greater odds of receiving heDMT (OR = 3.17; 95% CI, 1.41–8.13; *p* = 0.009), and the likelihood was markedly higher among patients treated between 2019 and 2024 (OR = 48.8; 95% CI, 21.4–128; *p* < 0.001). An initial clinical presentation with cerebellar symptoms was also independently associated with increased odds of initiating heDMT compared with sensory onset (OR = 2.51; 95% CI, 1.13–5.63; *p* = 0.025). No other variables, including sex, age, body mass index, comorbidities, or other clinical presentation, were significantly associated with first-line use of heDMT (Table 2).

**Table 2:** Determinants of heDMT based on multivariable logistic regression analysis.

**Characteristic**	**OR**	**95% CI**	***p*-Value**
**Gender**			
Male	Reference	Reference	
Female	0.79	0.49, 1.27	0.332
Age (years)	0.97	0.94, 1.00	0.065
**BMI**			
Underweight	Reference	Reference	
Normal	0.69	0.21, 2.22	0.535
Overweight	0.60	0.18, 1.96	0.404
Obese	0.76	0.23, 2.48	0.647
**Family history of MS**			
No	Reference	Reference	
Yes	0.65	0.35, 1.23	0.186
Unknown	1.07	0.64, 1.79	0.788
**Diabetes mellitus**			
No	Reference	Reference	
Yes	0.70	0.17, 2.59	0.607
**Hypertension**			
No	Reference	Reference	
Yes	1.05	0.27, 4.25	0.947
**Depression**			
No	Reference	Reference	
Yes	1.02	0.51, 2.02	0.962
**Thyroid disease**			
No	Reference	Reference	
Yes	0.50	0.20, 1.22	0.130
**Initial clinical presentation**			
Sensory	Reference	Reference	
Motor	1.60	0.84, 3.06	0.156
Cerebellar	2.51	1.13, 5.63	0.025
Brainstem	1.01	0.48, 2.17	0.976
Visual	1.30	0.72, 2.36	0.390
Others	1.27	0.30, 6.79	0.757
Time between MS diagnosis to initiation of therapy (months)	1.00	1.00, 1.01	0.984
**Era of DMT initiation**			
2008–2013	Reference	Reference	
2014–2018	3.17	1.41, 8.13	0.009
2019–2024	48.8	21.4, 128	< 0.001

CI, confidence interval; OR, odds ratio.

### Factors associated with current EDSS

In the multivariable ordinal logistic regression model evaluating factors associated with current EDSS disability status, starting on a heDMT was not associated with better or worse EDSS disability levels compared with starting on a platform therapy (OR = 1.08; 95% CI, 0.64–1.84; *p* = 0.767). Increasing age was independently associated with higher EDSS scores (OR = 1.08; 95% CI, 1.04–1.11; *p* < 0.001). Compared with underweight individuals, patients with normal (OR = 0.27; 95% CI, 0.09–0.86; *p* = 0.019), overweight (OR = 0.20; 95% CI, 0.07–0.66; *p* = 0.005), and obese BMI (OR = 0.18; 95% CI, 0.06–0.61; *p* = 0.003) categories had significantly lower odds of higher EDSS scores. Regarding clinical presentation, motor (OR = 3.93; 95% CI, 2.01–7.88; *p* < 0.001) and cerebellar symptoms (OR = 3.89; 95% CI, 1.71–8.78; *p* = 0.001) were strongly associated with higher disability categories compared with sensory onset (Table 3).

**Table 3:** Factors associated with current EDSS based on multivariable ordinal logistic regression analysis.

**Characteristic**	**OR**	**95% CI**	***p*-Value**
**DMT** ^*^			
Platform	Reference	Reference	
heDMT	1.08	0.64, 1.84	0.767
**Gender**			
Male	Reference	Reference	
Female	0.61	0.37, 1.02	0.059
Age (years)	1.08	1.04, 1.11	< 0.001
**BMI**			
Underweight	Reference	Reference	
Normal	0.27	0.09, 0.86	0.019
Overweight	0.20	0.07, 0.66	0.005
Obese	0.18	0.06, 0.61	0.003
**Family history of MS**			
No	Reference	Reference	
Yes	0.92	0.42, 1.90	0.829
Unknown	1.36	0.79, 2.32	0.254
**Diabetes mellitus**			
No	Reference	Reference	
Yes	1.22	0.39, 3.45	0.720
**Hypertension**			
No	Reference	Reference	
Yes	0.85	0.26, 2.50	0.779
**Depression**			
No	Reference	Reference	
Yes	1.08	0.53, 2.06	0.829
**Thyroid disease**			
No	Reference	Reference	
Yes	1.31	0.52, 3.05	0.545
**Initial clinical presentation**			
Sensory	Reference	Reference	
Motor	3.93	2.01, 7.88	< 0.001
Cerebellar	3.89	1.71, 8.78	0.001
Brainstem	1.99	0.81, 4.68	0.121
Visual	1.44	0.66, 3.08	0.352
Others	1.81	0.25, 8.22	0.486
Time between MS diagnosis to initiation of therapy (months)	1.00	1.00, 1.01	0.750

CI, confidence interval; OR, odds ratio.

^*^Primary exposure is first-line therapy class (heDMT vs Platform); all other variables are prespecified confounders.

### Risk of treatment discontinuation

**Figure 2:**
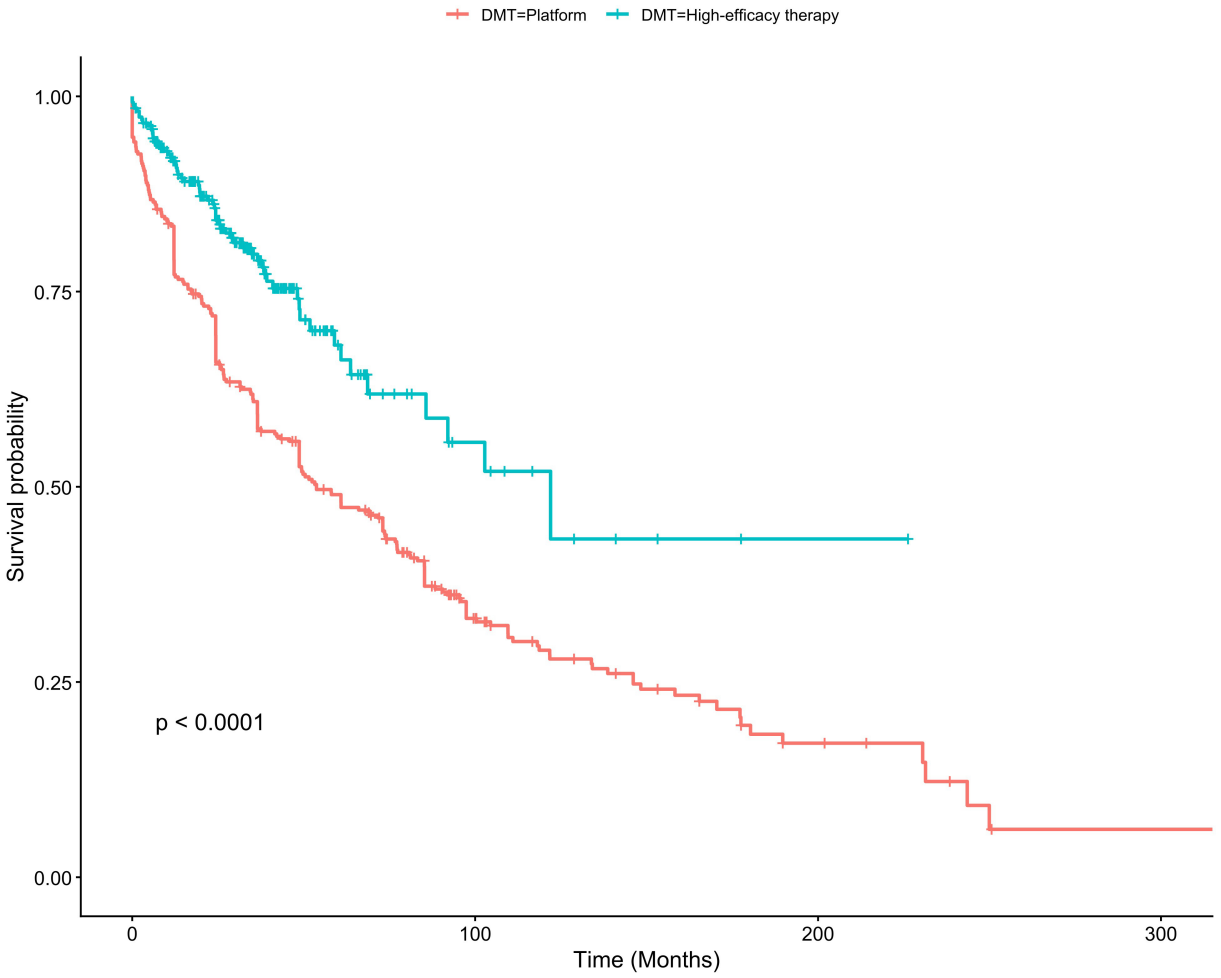
Kaplan–Meier estimates of treatment continuation over time across DMT groups. A significant difference in discontinuation risk was evaluated using the log-rank test (*n* = 589).

Kaplan–Meier analysis demonstrated a significantly higher probability of treatment persistence among patients who initiated heDMT compared to those who received platform therapy (log-rank *p* < 0.0001). At 12 months, the estimated treatment persistence rate was 91.7% (95% CI, 88.4%−95.2%) in the heDMT group vs. 83.4% (95% CI, 79.4%−87.5%) in the platform group. The survival curves diverged early and continued to separate over time, indicating sustained superior persistence among heDMT users throughout the follow-up period (Figure 2).

In the multivariable Cox proportional hazards model, initiation on heDMT was associated with a significantly lower hazard of treatment discontinuation compared to platform therapy (HR = 0.41, 95% CI, 0.30–0.57, *p* < 0.001). Increasing age was also associated with a lower risk of discontinuation (HR = 0.97, 95% CI, 0.96–0.99, *p* = 0.003). Additionally, the presence of thyroid disease was associated with a higher risk of discontinuation (HR = 1.71, 95% CI, 1.09–2.69, *p* = 0.020). No other variables were significantly associated with discontinuation risk (Table 4).

**Table 4:** Results of the multivariable cox proportional model for the risk of treatment discontinuation (*n* = 589)^*^.

**Characteristic**	**HR**	**95% CI**	***p*-Value**
**DMT** ^†^			
Platform	Reference	Reference	
heDMT	0.41	0.30, 0.57	< 0.001
**Gender**			
Male	Reference	Reference	
Female	1.15	0.86, 1.53	0.335
Age (years)	0.97	0.96, 0.99	0.003
**BMI**			
Underweight	Reference	Reference	
Normal	0.80	0.43, 1.49	0.477
Overweight	0.92	0.49, 1.72	0.796
Obese	0.69	0.36, 1.31	0.256
**Family history of MS**			
No	Reference	Reference	
Yes	0.76	0.52, 1.11	0.162
Unknown	0.81	0.60, 1.09	0.160
**Diabetes mellitus**			
No	Reference	Reference	
Yes	0.56	0.28, 1.11	0.096
**Hypertension**			
No	Reference	Reference	
Yes	1.31	0.60, 2.86	0.500
**Depression**			
No	Reference	Reference	
Yes	1.30	0.93, 1.83	0.126
**Thyroid disease**			
No	Reference	Reference	
Yes	1.71	1.09, 2.69	0.020
**Initial clinical presentation**			
Sensory	Reference	Reference	
Motor	0.90	0.63, 1.27	0.531
Cerebellar	1.08	0.68, 1.72	0.746
Brainstem	1.23	0.80, 1.88	0.348
Visual	0.82	0.58, 1.17	0.271
Others	0.75	0.23, 2.45	0.633
Time between MS diagnosis to initiation of therapy (months)	1.00	1.00, 1.01	0.250

^*^The analysis included all records with non-missing time-to-event and event status information (*n* = 589).

^†^Primary exposure is first-line therapy class (heDMT vs Platform); all other variables are prespecified confounders.

CI, confidence interval; HR, hazard ratio.

### Cause-specific analysis of treatment discontinuation

In the Fine–Gray competing-risks regression model evaluating specific causes of treatment discontinuation, initiation with heDMT was associated with significantly lower sub distribution hazard ratios for discontinuation due to inefficacy (sHR = 0.12, 95% CI, 0.05–0.32, *p* < 0.001) and adverse events (sHR = 0.22, 95% CI, 0.12–0.38, *p* < 0.001) when compared to platform therapies. In contrast, heDMT was associated with a significantly higher hazard of discontinuation due to other reasons, such as pregnancy, preference, or supply issues (sHR = 3.46, 95% CI, 2.68–4.46, *p* < 0.001). Increasing age was independently associated with a lower hazard of discontinuation due to adverse events (sHR = 0.97; 95% CI, 0.95–1.00; *p* = 0.030) and other causes (sHR = 0.99; 95% CI, 0.97–1.00; *p* = 0.042). Additionally, patients with an initial motor presentation were less likely to discontinue therapy for other reasons (sHR = 0.69; 95% CI, 0.50–0.96; *p* = 0.026; Table 5).

**Table 5:** Results of competing-risks (Fine–Gray) for treatment discontinuation (*n* = 519)^*^.

**Characteristic^§^**	**Inefficacy**			**Adverse events**			**Others**		
	**sHR**	**95% CI**	***p*** **-Value**	**sHR**	**95% CI**	***p*** **-Value**	**sHR**	**95% CI**	***p*** **-Value**
heDMT	0.12	0.05, 0.32	< 0.001	0.22	0.12, 0.38	< 0.001	3.46	2.68, 4.46	< 0.001
Female	0.69	0.38, 1.26	0.230	0.90	0.60, 1.34	0.600	1.14	0.91, 1.42	0.250
Age (years)	1.03	1.00, 1.07	0.073	0.97	0.95, 1.00	0.030	0.99	0.97, 1.00	0.042
BMI–normal	1.52	0.19, 12.4	0.700	0.73	0.25, 2.12	0.560	1.34	0.69, 2.61	0.380
BMI–overweight	1.57	0.19, 13.0	0.670	0.83	0.28, 2.41	0.730	1.43	0.73, 2.78	0.300
BMI–obese	1.67	0.20, 13.8	0.630	0.58	0.20, 1.73	0.330	1.56	0.80, 3.04	0.190
Diabetes mellitus	0.25	0.03, 1.91	0.180	2.05	0.96, 4.40	0.065	0.84	0.48, 1.49	0.550
Hypertension	0.59	0.16, 2.20	0.440	1.83	0.63, 5.34	0.270	0.84	0.43, 1.65	0.620
Depression	1.13	0.53, 2.38	0.760	1.15	0.65, 2.01	0.640	0.80	0.54, 1.18	0.270
Thyroid disease	0.67	0.24, 1.86	0.440	1.08	0.52, 2.24	0.840	1.00	0.58, 1.71	0.990
Family history of MS–yes	1.34	0.67, 2.66	0.400	0.78	0.42, 1.46	0.440	0.98	0.72, 1.33	0.890
Family history of MS–unknown	0.64	0.31, 1.29	0.210	0.78	0.50, 1.21	0.260	1.25	0.98, 1.59	0.076
Presentation–motor	1.22	0.60, 2.49	0.580	1.45	0.87, 2.40	0.150	0.69	0.50, 0.96	0.026
Presentation–cerebellar	1.05	0.36, 3.06	0.920	0.88	0.41, 1.89	0.730	0.89	0.59, 1.34	0.570
Presentation–brainstem	1.03	0.39, 2.73	0.950	1.00	0.52, 1.90	0.990	1.03	0.71, 1.48	0.890
Presentation–visual	0.87	0.38, 1.96	0.730	0.81	0.46, 1.42	0.450	0.97	0.73, 1.31	0.860
Presentation–others	1.23	0.15, 10.1	0.850	NE	NE	NE	2.60	1.65, 4.10	< 0.001
Time between MS diagnosis to initiation of therapy (months)	1.00	0.99, 1.01	0.650	1.00	0.98, 1.01	0.380	1.00	1.00, 1.00	0.002

^§^Inefficacy (labels including failure/non-response and disease activity/progression), adverse events (labels including side effect and tolerance/intolerance), and others (labels including pregnancy planning, patient preference, lack of supply, and not known).

^*^For the competing-risks Fine–Gray regression, a complete-case approach was used because the multivariable models adjust for additional covariates; records with missing data in any of the following were excluded: DMT, gender, age, BMI, diabetes, hypertension, depression, thyroid disease, family history of MS, initial clinical presentation, and time from diagnosis to DMT initiation. This yielded 519 analyzable subjects.

NE: non-estimable. No events in this stratum; model could not estimate an effect.

CI, confidence interval, sHR, subdistribution hazard ratios.

## Discussion

This study provides new real-world evidence on factors influencing the initial selection of heDMTs for relapsing-remitting multiple sclerosis (RRMS) in Saudi Arabia and on treatment persistence across therapeutic classes. We found that the use of heDMTs as first-line disease-modifying therapies (DMTs) increased dramatically in recent years, reflecting a marked shift toward early intensive management. Nearly 40% of newly treated patients initiated therapy with heDMT, significantly higher than earlier international reports, where first-line heDMT use was closer to one-quarter of newly treated cases. This change in practice reflects a broader global trend toward early, high-efficacy treatment strategies in multiple sclerosis, increasingly favoring potent oral and infusion-based agents (1, 10–12).

The strong temporal association between treatment era and heDMT initiation underscores the growing physician confidence and accessibility of these therapies. Monoclonal antibody-based agents such as ocrelizumab and rituximab have become increasingly favored in Saudi Arabia as their tolerability and real-world effectiveness profiles have matured. Evidence from large-scale studies indicates that patients who initiate heDMT early experience significantly lower relapse rates, reduced treatment switching, and higher rates of no evidence of disease activity (NEDA) compared with those managed through an escalation approach (13–15). These findings are consistent with prior meta-analyses showing that treatment initiation of DMTs, irrespective of treatment class, within the first year of MS onset is associated with slower disability accumulation and improved long-term outcomes (16). Thus, the rapid adoption of these therapies within the Kingdom reflects not only shifting attitudes among neurologists but also improved therapeutic availability and alignment with evidence-based practice.

Interestingly, our findings indicated that presentation with cerebellar symptoms was a significant predictor of initiating a heDMT. Neurologists may interpret cerebellar involvement at onset as markers of potentially aggressive disease, warranting strong early disease suppression. Similar trends have been noted in studies from Europe and North America, where heDMTs are preferentially prescribed for patients with high MRI lesion load, frequent relapses, or poor recovery profiles. Other demographic or comorbidity factors (such as age, sex, or BMI) did not significantly influence the likelihood of receiving heDMT, suggesting that clinical phenotype and evolving prescription eras were the primary determinants (17).

Although the first-line use of heDMTs was not significantly associated with current disability status (EDSS) in our cohort, this likely reflects the relatively short disease duration and the predominance of mildly disabled patients. Age emerged as a major factor linked with higher disability levels, consistent with prior registries indicating that neurodegeneration accelerates with age regardless of treatment class. The observed inverse relationship between higher BMI and disability could reflect residual confounding or reverse causation, as weight loss often accompanies more advanced disease rather than being protective (16).

The persistence analyses demonstrated a robust advantage for heDMT. Patients who began treatment with a heDMT remained on their initial therapy longer and were less likely to discontinue

due to inefficacy or adverse events. These findings are consistent with previous real-world studies showing that persistence reflects both superior efficacy and tolerability of modern heDMTs such as ocrelizumab, cladribine, and ofatumumab. A pooled analysis of ocrelizumab extension studies reported approximately 90% treatment continuation at 2 years, paralleling our own Kaplan-Meier estimates of sustained persistence in the heDMT group. In contrast, older platform agents like interferons were more prone to discontinuation for lack of efficacy or tolerability issues, including flu-like symptoms, injection-site reactions, and laboratory abnormalities. This pattern has also been reported across the MSBase and CONFIDENCE cohorts (18–20).

Concerns regarding the timing of heDMT initiation were addressed analytically by adjusting for time from diagnosis to treatment initiation across all multivariable models. In our cohort, this variable was not independently associated with disability status or treatment discontinuation, suggesting that the observed differences in persistence and outcomes were not primarily driven by delayed treatment initiation. Nonetheless, we acknowledge that more granular categorization of early vs. delayed heDMT initiation may provide additional insights and warrants further study.

An additional layer of nuance comes from our competing-risks analysis, which revealed that heDMT users were less likely to discontinue for medical reasons but more likely to stop treatment for “other” causes such as pregnancy, patient preference, or supply limitations. These results highlight not only the superior pharmacological performance of high-efficacy agents but also the practical factors influencing their long-term use, particularly among younger female patients or those navigating treatment access and monitoring challenges. The finding that older patients were less likely to discontinue reinforces prior observations that younger individuals tend to switch therapy more frequently in pursuit of lifestyle flexibility or reduced monitoring burden (6, 21). The higher rate of discontinuation for non-medical reasons among patients initiating heDMTs was largely driven by pregnancy-related treatment interruption. Importantly, this finding should be interpreted in the context of local prescribing practices during the study period rather than as an inherent limitation of high-efficacy agents. While pregnancy has traditionally prompted discontinuation of disease-modifying therapies in many settings, emerging international evidence and expert consensus now support the continued use of selected agents, particularly natalizumab and anti-CD20 therapies such as ocrelizumab, under carefully defined conditions and individualized risk-benefit assessment, often in off-label clinical contexts (22, 23).

Beyond clinical characteristics, treatment selection in our cohort was likely shaped by health system-level and logistical factors. In Saudi Arabia's publicly funded healthcare system, high-cost disease-modifying therapies are centrally approved and provided without direct patient cost, which may attenuate affordability barriers commonly encountered in insurance-based systems. Within this context, anti-CD20 therapies, particularly ocrelizumab, which accounted for 56.4% of first-line heDMT use, appear preferentially selected due to their robust efficacy, favorable tolerability, infrequent dosing, and lower monitoring burden. This aligns with contemporary real-world prescribing trends in which anti-CD20 agents have become the dominant first-line high-efficacy option (24). However, the disproportionate representation of ocrelizumab in our cohort may introduce heterogeneity within the high-efficacy group, and outcomes should therefore be interpreted as reflecting class-level patterns rather than agent-specific comparative effectiveness.

From a broader perspective, our study captures the evolving therapeutic paradigm in Saudi Arabia, which is increasingly convergent with global best practices advocating early, proactive management of MS. The growing preference for initiating potent agents aligns with contemporary evidence highlighting that early control of inflammatory disease activity translates into better long-term outcomes, including delayed conversion to secondary progressive MS and sustained independence. Since registry data suggest that the efficacy of DMTs diminishes with age, broadening access to heDMTs at earlier disease stages may significantly alter the national trajectory of MS disability. This shift also reflects a response to local expert consensus that early use of heDMTs remains underutilized in the Kingdom due to risk aversion, monitoring demands, and logistic factors (8, 9, 18).

### Limitations

Despite the strengths of a large, well-defined cohort, several limitations should be acknowledged. First, the retrospective, single-center design limits generalizability, as patients treated in tertiary referral settings often have higher disease activity and/or prior treatment failure. This may have inflated the observed rate of first-line heDMT use relative to the broader population. Second, reliance on routine clinical documentation introduces potential variability in data quality, and we lacked complete information on adherence and socioeconomic factors, which may have influenced treatment selection and outcomes. Disability was assessed using the EDSS; although standardized and widely used, the EDSS is weighted toward ambulatory function and is less sensitive to cognitive impairment, fatigue, and quality-of-life domains. As with all observational studies, causal relationships between treatment category and outcomes cannot be established, and residual confounding remains possible. In addition, we did not perform a prespecified subgroup analysis comparing early vs. delayed initiation of heDMT, and confounding related to treatment timing cannot be fully excluded. Pregnancy-related discontinuation patterns may also have been affected by evolving evidence and changing guidance regarding the safety of certain high-efficacy agents during pregnancy over the study period, limiting direct comparisons with contemporary international practice. Finally, treatment choice may have been shaped by system-level factors, including centralized drug procurement in a publicly funded healthcare system. The predominance of ocrelizumab within the heDMT group may also have introduced treatment-mix imbalance, such that observed outcomes largely reflect one agent rather than the entire heDMT class. Accordingly, findings should be interpreted at the class level and may not be generalizable to settings with different prescribing practices or for agent-specific comparisons.

### Future research directions

Future research should extend these findings through multicenter prospective designs and stronger integration with national MS registry platforms. Broader geographic sampling would help capture variations in prescribing behavior between tertiary, secondary, and peripheral centers, as well as between public and private health systems. Longitudinal followup- is essential to evaluate durability of efficacy, cognitive trajectories, and the long-term safety of early heDMT use, including the impact of early vs. delayed initiation on disability progression and treatment persistence. Incorporating biomarkers such as neurofilament light chain (NfL) and MRI-based parameters could refine response prediction and guide therapy sequencing. Further, mixed methods work that captures patient perceptions, physician attitudes, and institutional barriers would add context to quantitative trends, helping design targeted interventions that encourage equitable- access to heDMTs nationwide.

## Conclusion

heDMTs are increasingly being adopted as first-line options for multiple sclerosis treatment in Saudi Arabia, reflecting a noticeable departure from the historic escalation model toward early intensive disease control. Their use is most common in patients presenting with cerebellar features and in those diagnosed during more recent treatment eras, indicating a growing awareness among physicians of the prognostic importance of early aggressive therapy. Patients who received high-efficacy agents achieved better treatment persistence and lower discontinuation due to inefficacy or tolerability issues compared to those starting with platform therapies.

These findings reinforce international evidence supporting early initiation of potent DMTs to optimize long-term neurological outcomes. At the same time, they show the continued need to address local disparities in access, cost, and infrastructure that can hinder equitable treatment implementation. The integration of real-world data from national MS registries, together with sustained professional education, could further enhance early decision-making and ensure that eligible patients benefit from timely high-efficacy therapy.

Ultimately, the shift documented in this study represents more than a change in prescribing pattern. It signals a maturing therapeutic culture that aligns with evolving scientific evidence and prioritizes long-term stability and quality of life for people living with MS. Expanding this approach through coordinated policy, patient-centered counseling, and multicenter research collaboration will be critical to consolidating these gains and shaping the next chapter of MS management in the Kingdom.

## Data Availability

The raw data supporting the conclusions of this article will be made available by the authors, without undue reservation.
